# Biting Hour and Host Seeking Behavior of *Aedes* Species in Urban Settings, Metema District, Northwest Ethiopia

**DOI:** 10.3390/tropicalmed10020038

**Published:** 2025-01-28

**Authors:** Wondmeneh Jemberie, Sisay Dugassa, Abebe Animut

**Affiliations:** 1 Vector Biology & Control Research Unit, Aklilu Lemma Institute of Pathobiology, Addis Ababa University, Addis Ababa P.O. Box 1176, Ethiopia; sisay.dugassa@aau.edu.et (S.D.); abebe.animut@aau.edu.et (A.A.); 2 Department of Biology, College of Natural and Computational Sciences, University of Gondar, Gondar P.O. Box 196, Ethiopia

**Keywords:** *Aedes* mosquito, biting hour, host seeking, urban area

## Abstract

Background: *Aedes* species transmit arboviral diseases, such as dengue, chikungunya, yellow fever, and Zika. The diseases cause severe sickness, mortality, and economic losses. This study describes the biting hour and host-seeking behavior of *Ae. aegypti* and *Ae. vittatus* in three towns. Recently, chikungunya and dengue infections were reported in the study sites. Methods: Biting hour and host-seeking behaviors of *Ae. aegypti* and *Ae. vittatus* were studied from June to September 2023, in Genda-Wuha, Kokit, and Metema-Yohannes towns, Metema district, Northwest Ethiopia. CDC-LT traps were set running indoors and outdoors for 24 h closer to humans sleeping inside unimpregnated mosquito nets. At the same time, CDC-LT traps were set running overnight closer to domestic animals’ shelters located within a 50-m radius of the main residence. Mosquitoes trapped in CDC-LT were collected every hour. The study was conducted four times in each town during the wet season. A chi-square test was employed to examine biting hour and host-seeking behavior. Results: *Aedes aegypti* was observed to be highly exophilic and active during the daylight hours. *Aedes aegypti* exhibited a peak biting rate between 07:00 and 08:00 with the biting rate of 4.5/person/hour followed by from 17:00 pm to 18:00 pm with the biting rate of 3.75/person/hour. The hourly biting rate of *Ae. aegypti* differed significantly. Its peak indoor biting rate was from 19:00 to 20:00 with the rate of 2.00 bites/person/hour followed by from 08:00 to 09:00 with the rate of 1.50 bites/person/hour and the biting rates differed significantly across the hours (F = 240.046; *p* = 0.001). *Aedes vittatus* also exhibited a biting rate similar to that of *Ae. aegypti*. Both *Ae. aegypti* and *Ae. vittatus* were abundantly collected from nearby human sleeping arrangements than from the shelters of cattle, sheep, goats, and donkeys. The highest proportions of *Ae. aegypti* (91.21%) and *Ae. vittatus* (89.87%) were unfed. Conclusions: *Aedes aegypti* and *Ae. vittatus* exhibited peak biting rates during morning and early night hours that aligned with the active daily routine practices of the local community. This could potentially expose the inhabitants to viral diseases transmitted by *Ae. aegypti* and *Ae. vittatus*.

## 1. Introduction

Mosquitoes in the genus *Aedes* transmit viral pathogens that cause yellow fever (YF), dengue (DEN), chikungunya (CHIK), and Zika (ZIK) to humans through their bites [1,2]. Dengue is transmitted globally, causing approximately five million infections and more than 5000 deaths in over 80 countries annually [3,4] and Yellow Fever (YF), which is mainly a problem in Africa [5] and South America [6], causes over 200,000 global cases and 30,000 deaths [7]. The expansion of arboviral disease distribution is also attributed to the movement of infected hosts and mosquito invasions facilitated by global changes, trade of goods, and travel [8,9]. Furthermore, changes in the human environment due to urbanization, globalization, and greater human mobility contributed to the rapid growth in arboviral infections [10]. However, governments and scientific institutions pay little attention to the diseases.

In the past year, unexpected epidemics of viral infections such as dengue fever occurred in eastern, southeastern, southern, and northwestern Ethiopia [11,12,13]. Chikungunya cases were documented in Ethiopia, Sudan [14] and Kenya [15], and for the first time, an outbreak was reported from Suuf village in the Dollo Ado district, Somalia region, Ethiopia [16,17]. Furthermore, Ethiopia is one of Africa’s countries where the yellow fever virus is transmitted [18]. A previous study found that yellow fever epidemics occurred in South Omo villages in Ethiopia [19], as well as in the Gurage Zone in southwestern Ethiopia [20].

Some people move seasonally from the highlands to the Metema district and back. Similarly, Sudanese people come from endemic areas of arboviral diseases in east Sudan to visit the district briefly for the trade system [21,22]. These visitors sleep outdoors and do not own nets to sleep in. In the district, during the dry season, most people sleep outside of their homes due to high temperatures, pressure, and lack of air conditioning and ventilation systems inside their homes. Such outdoor sleeping arrangements favor uninterrupted biting activity for *Aedes* species and hence lead to the transition of viral diseases from infected to susceptible individuals in the area and also the establishment of transmission of the diseases in the highlands. Outdoor and indoor sleeping arrangements in the absence of effective mosquito control strategies, including the absence of mosquito repellents, limitation of consistent larvae intervention, and limited access to long-lasting insecticide-treated nets, favor a high biting rate of *Aedes* species in the study sites [23].

*Aedes* species have a variety of resting and larval habitats [24], diverse blood meal sources [25], the ability to be evolved into insecticide resistant, and biting behaviors that occur during the day and at night [26]. All of these characteristics contribute to the distribution and vectorial of *Aedes* species [27]. These behaviors heavily contribute to their distribution, abundance, and disease transmission capacity. They can comprise anthropophilic, endophilic, exophagic, and endophagic behaviors. Females of *Ae. aegypti* regularly and preferentially feed on human blood [28,29]. However, their host selection is determined by host availability and ambient weather condition, such as humidity, temperature, and light intensity [30].

*Aedes aegypti* and *Ae. vittatus* breed in artificial habitats in the wet season in the study towns [24]. Recent serological tests detected chikungunya virus [31] and dengue virus infections [32] among febrile cases that attended outpatient departments of health centers in the Metema District. Despite the public health implications and recent outbreaks of arboviral diseases, there is limited information on the behavior and seasonal dynamics of *Aedes* species in the district. The study described biting hour, host-seeking behavior, and physiological status that impacted the biting rate of *Ae. aegypti* and *Ae. vittatus* in the Metema district, Northwest Ethiopia.

## 2. Study Setting and Methods

### 2.1. Study Setting

**Figure 1:**
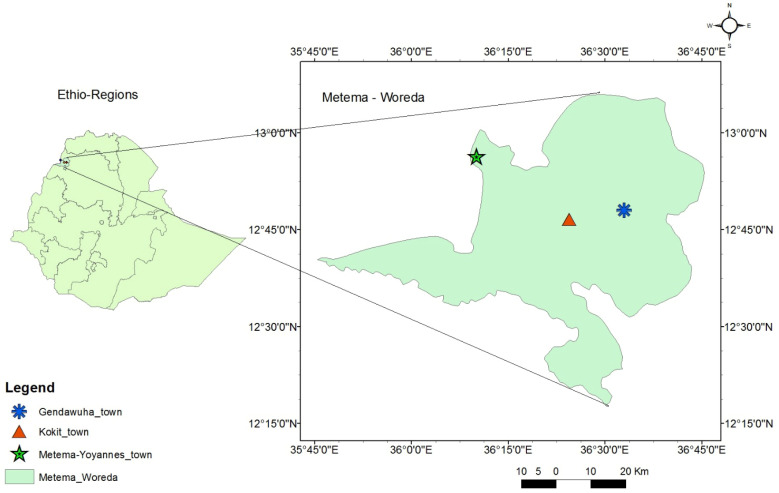
Map of Genda-wuha, Kokit, and Metema-Yohannes towns, Metema district, Northwest Ethiopia. Source: Ethio_GIS, 2023.

The study was conducted in Metema-Yohannes (12.950649 N, 36.147112 E), Kokit (12.822325 N, 36.326846 E), and Genda-Wuha (12.822333 N, 36.326861 E) towns, Metema district, Ethiopia. The district is 180 km west of Gondar and 925 km northwest of Addis Abeba (Figure 1). The rainy season lasts from June to September, and the average temperature is 25–30 °C. The dry season, October to May, has higher average temperatures (28–42 °C). The district has an average elevation of 734 m above sea level and gets a unimodal rainfall with an annual precipitation of 500–800 mm [33]. There were reports of yellow fever, chikungunya, and dengue cases at the health facilities and along the border with East Sudan [22,34].

### 2.2. Study Design

**Figure 2:**
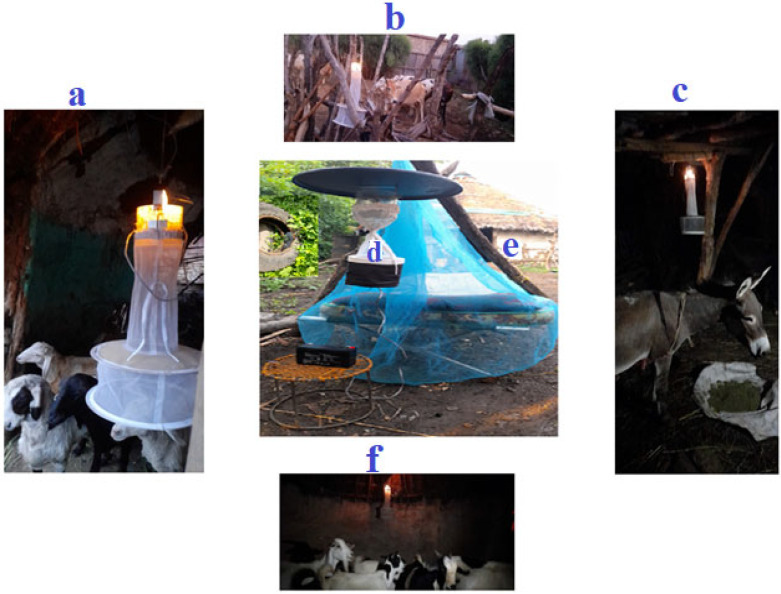
Collection of *Aedes* species using CDC light trap within a 50 m radius from the target house. (**a**) sheep, (**b**) cattle, (**c**) donkey, (**d**) outdoor (human), and (**e**) indoor (human), (**f**) goat.

Biting hour and host-seeking behavior of adult female *Aedes* species were assessed in three towns. Ten households were selected from each town based on certain criteria, such as the presence of larvae-holding containers and the availability of at least four domestic animals within the compound. Data were collected from June to September 2023. Female *Aedes* species that attempted to bite humans (indoors and outdoors) were trapped using a CDC light trap (CDC-LT) (Model 512 (John W. Hock Company, Gainesville, FL, USA), operating close to a man sleeping under an insecticide-free mosquito net every hour for 24 h (daylight hour: 06:00–18:00, night hour: 18:00–06:00). The collection was made for three consecutive days per month. In the same time, female *Aedes* species attempting to feed on cattle, sheep, goats, and donkeys in shelters within a 50-m radius of the houses were trapped overnight (18:00–06:00) CDC-LT [35] (Figure 2). The trapped mosquitoes were put into prelabeled holding cups.

### 2.3. Identification of Mosquito Species

Female mosquitos were initially recognized by species using the Leopoldo identification keys under a Zeiss Stemi 508 stereo microscope (Carl Zeiss AG, Oberkochen, Germany) [35]. Their morphological characteristics include white scales on the occipital and vertex of the head, white spots and stripes on the scutum, presence of bands in the abdomen, presence or absence of a white band on the palp, and the banding and claws on the legs, which are distinguished by pale and spirally stripes on the tarsus and tarsi claws [36] (see Supplementary Materials Figure S1). Each Aedes species was preserved separately in a perforated 1.5-mL microcentrifuge tube and stored in a zip-lock plastic bag containing silica gel for subsequent molecular confirmation.

To confirm *Ae. aegypti* and *Ae. vittatus*, DNA was extracted from the legs of each preserved specimen using Phenol-Chloroform-Isoamyl alcohol technique [37]. Polymerase chain reaction (PCR) was used to target the mitochondrial cytochrome oxidase subunit 1 (COI) and internal transcribed spacer 1 (ITS1) genes in Aedes aegypti and Aedes vittatus, respectively. A 735 bp area bordering the mitochondrial COI gene was amplified by polymerase chain reaction (PCR) with the following primers: forward 5- GGATTTGGAAATTGATTAGTTCCTT-3 and reverse 5-AAAAATTTT AATTCCAGTTGGAACAGC-3. Each amplification was conducted in 20 µL of 1× GoTaq Green Master Mix (Promega, Fitchburg, WI, USA), 3 µL of DNA template, and 0.5 µL of forward and reverse primers (10 pmol). The sample was heated to 95 °C for 5 min before being subjected to 35 cycles of 94 °C for 30 s, 56 °C for 30 s, and 72 °C for 45 s, followed by a final extension step of 72 °C for 10 min. The second gene is a 700 bp region bordering internal transcribed spacer 1 (ITS1) with the primers: forward 5-GAAGTAAAAGTCGTAACAAGG-3 and reverse 5- CGACCCTCAGACAGGCGTGGC-3. The PCR amplification was performed with a thermocycler (Veriti, Applied Bioosystems, Taipei, Taiwan). Each 20 µL reaction mixture contained 0.5 µL DNA forward and reverse primers, 2.5 µL 10× PCR buffer (Mg^2+^), 2 µL dNTP mixture (10 mM each), 1 unit of Taq DNA polymerase, and an adequate volume of molecular grade water. For comparison, appropriate amounts of sterile distilled water were added to the reaction mixture to serve as a negative control. The PCR conditions included denaturation at 94 °C for 5 min, followed by 35 cycles of denaturation at 94 °C for 1 min, annealing at 53 °C for 1 min, extension at 72 °C for 1 min, and final extension at 72 °C for 10 min. The third step was setting the temperature to 4 °C for an infinite period of time [38]. 

Polymerase chain reaction (PCR) products were observed in a 1.5% agarose gel electrophoresis using UV light. The specimens that tested positive for Aedes aegypti and Aedes vittatus were transported to Macrogen Biotechnology Company, Ltd. in South Korea, where they were sequenced using Sanger. Multiple aligned with BioEdit Sequence Alignment Editor and deposited in the GenBank database.

### 2.4. Data Analysis

Data were coded and analyzed using IBM SPSS statistical software for Windows (IBM corp., Armonk, NY, USA), version 20.0. Comparison of counts and proportions of fed, half-gravid, and gravid made among towns, animals, and collection sites. Data were checked by the Shapiro–Wilk test for being normally distributed, and those not normally distributed were log-transformed [log(n + 1)] to fit the normal distribution curve and analyzed by one-way ANOVA using Tukey Kramer multiple comparison with the post hoc *t*-test. The biting rates were compared among the study sites, biting hours, host groups, and abdominal status of female Aedes mosquitoes. The independent sample t-test was used to compare Aedes species biting rates between two biting hours in both outdoor and indoor setting. Mosquito biting rate (MBR) is determined as the total number of female *Ae. aegypti* or *Ae. vittatus* per person per hour. Statistical values were considered significant when corresponding *p* < 0.05. Phylogenetic analysis was performed for selected *Ae. aegypti* and *Ae. vittatus* samples, in which case their gene sequences were aligned with CLUSTAL W software version 1.6, examined using the program MEGA (Molecular Evolutionary Genetics Analysis) version 7.1.16 Neighbor joining (NJ) and their phylogenetic trees were constructed.

## 3. Results

### 3.1. Composition of Mosquito Species

A total of 16,324 female mosquitos were collected from three study towns, of which 42.3% (n = 6915) were from Metema-Yohannes, 24.1% (n = 3946) from Kokit, and 33.4% (n = 5463) from Genda-Wuha town (Table 1). There was no significant difference in the total number of mosquito catches among the towns (F = 0.386; df = 2.00; *p* = 0.695). The mosquitoes comprised the genus *Culex* (55.4%; n = 9056), *Aedes* (39.2%; n = 6411), *Anopheles* (4.9%; n = 800), and *Culiseta* (0.3%; n = 57). Among the 6411 *Aedes* mosquitoes, 67.5% (n = 4333) were *Ae. aegpti*, 29.5% (n = 1895) *Ae. vittatus*, 1.6% (n = 107) *Ae. communis*, 0.7% (n = 48) *Ae. albopictus*, and 0.4% (n = 28) *Ae. opok*. Similarly, the 800 anophelines were composed of 60.2% (n = 482) of *An. gambiae* s.l., 25.8% (n = 207) of *An. stephensi*, 8.7% (n = 70) of *An. pretoriensis*, 3.0% (n = 24) of *An. christyi* and 2.1% (n = 17) of *An. cinereus*. Among all the mosquitoes (n = 16,324) caught by the CDC-LT, 79.5% (12,492) were caught during night hours and 23.4% (3822) during the daylight hour. All the *Anopheles*, *Culex*, and *Culiseta* were caught during night hours, whereas *Aedes* species were caught during both night and daylight hours.

### 3.2. Hourly Human Biting Behavior of Aedes aegypti and Aedes vittatus

**Figure 3:**
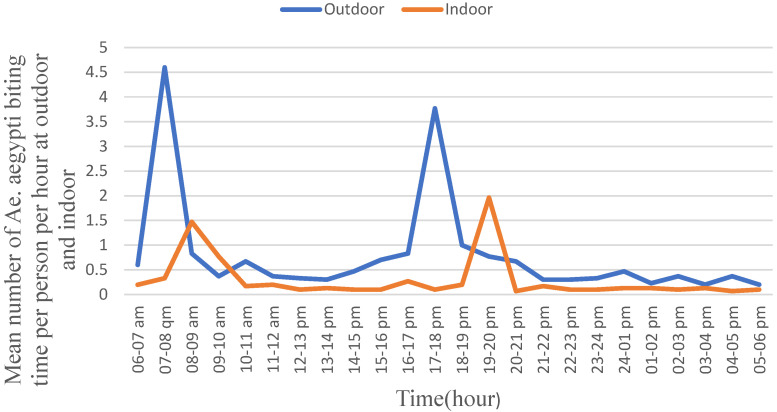
Human biting behavior of *Aedes aegypti*, Metema District, Northwest Ethiopia, June–September 2023.

In general, the outdoor biting rate of *Ae. aegypti* was higher than its indoor activity during both the daylight hour and night hour (Figure 3). Moreover, during the daylight hour, *Ae. aegypti* exhibited a peak biting rate between 07:00 and 08:00 with the biting rate of 4.5/person/hour followed by from 17:00 to 18:00 with the rate of 3.75/person/hour. The biting rates differed significantly across the collection hours (F = 480.630; *p* = 0.001). The peak indoor biting rate of *Ae. aegypti* was observed from 19:00 to 20:00 with a biting rate of 2.00 bites/person/hour followed by from 08:00 to 09:00 with the biting rate of 1.50 bites/person/hour. The indoor biting rates differed significantly (F = 240.046; *p* = 0.001) across the hours. Moreover, its peak indoor biting activity in the morning hours was late by one hour compared to the corresponding peak outdoor biting hours, and the values did not differ significantly.

**Figure 4:**
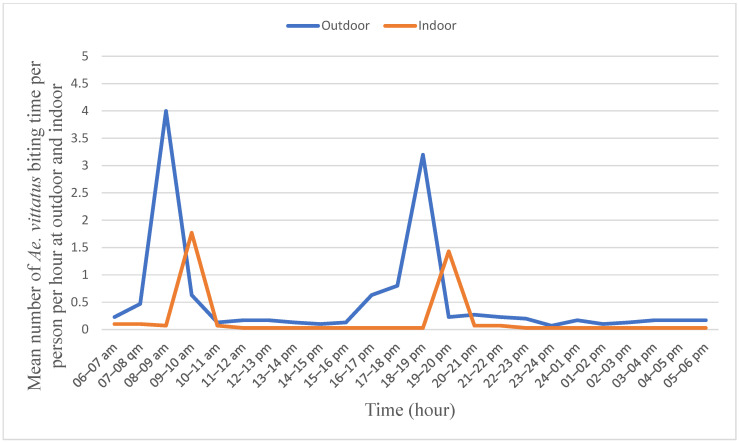
Human biting behavior of *Aedes vittatus* Metema District, Northwest Ethiopia, June–September 2023.

The outdoor peak biting rate of *Ae. vittatus* was observed from 08:00 to 09:00 in the morning with 4.00 bites/person/hour followed by from 18:00 to 19:00 in the afternoon with 3.25 bites/person/hour (Figure 4). Similarly, its peak indoor biting rate was observed between 09:00 and 10:00 in the morning with a bite rate of 1.75/hour/person followed by from 19:00 to 20:00 in the afternoon with a rate of 1.50 bites/person/hour. The biting rates differed significantly (F = 217.752; *p* = 0.001) across the hours. However, no significant difference was observed between two consecutive peaks outdoors and also indoors.

### 3.3. Host Seeking Behavior and Physiological Status of Aedes aegypti and Aedes vittatus

A total of 6411 female *Aedes* mosquitoes were collected using CDC-LT in the towns, of which 79.12% (4928/6411) were from the households (indoor and outdoor) with human stimulants and 20.87% (1300/6411) from domestic animal (cattle, sheep, goat, and donkey) shelters located in the compound. The highest percentage (81.62%) of *Ae. aegypti* preferred human, followed by cattle (6.92%), sheep (4.73%), goat (3.87%), and donkey (2.83%). The highest number of *Ae. aegypti* preferred human hosts as blood meal sources, and it showed a significant difference (*p* < 0.001) compared to other hosts in the study sites (Table 2). Similarly, the highest number of *Ae. vittatus* was collected from nearby humans (73.40%), followed by cattle (11.34%), sheep (6.22%), goats (4.85%), and donkeys (4.16%) with a significant difference (*p* < 0.001).

The highest proportion of *Ae. aegypti* was unfed (91.21%), followed by the blood-fed (6.20%) and gravid (2.58%). There was a significant difference (*p* < 0.001) among the abdominal status of *Ae. aegypt*. Similarly, the highest proportion of *Ae*, *vitatus* was unfed (89.87%), followed by the blood-fed (7.86%) and gravid (2.27%) physiological conditions. There was also a significant difference (*p* < 0.001) among the abdominal status of female *Ae. vittatus*.

### 3.4. Molecular Identification of Aedes aegypti and Aedes vittatus

**Figure 5:**
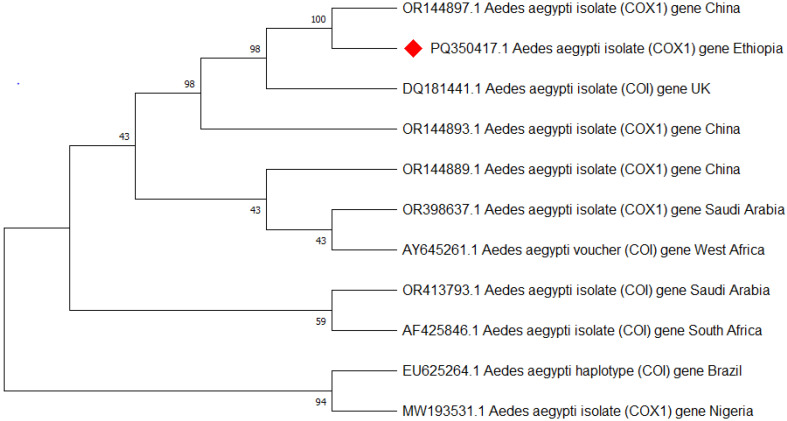
Phylogenetic tree representing the *Aedes aegypti* COI gene of the Ethiopian sequence is indicated with a red color. The evolutionary history was inferred using the neighbor-joining method [40]. The bootstrap consensus tree inferred from 500 replicates [41] and the percentage of replicate trees in which the associated taxa clustered together in the bootstrap test (500 replicates) are shown next to the branches [41]. The evolutionary distances were computed using Tamura–Kumar method [42]. This analysis involved 11 nucleotide sequences. All positions containing gaps and missing data were eliminated. There was a total of 554 positions in the final dataset. Evolutionary analyses were conducted in MEGA11 [43].

Approximately 25 of the morphologically identified *Ae. aegypti* were analyzed for species confirmation using PCR targeting the mitochondrial COI gene, and all were confirmed to be *Ae. aegypti*. Then after three of the *Ae. aegypti* (one from each town) that showed better bands were sequenced, edited, and aligned using the basic local alignment search tool (BLAST) analysis [39] and deposited in GenBank with the accession code of *Ae. aegypti* isolate AEAEM1 (PQ350417.1) (Figure 5).

**Figure 6:**
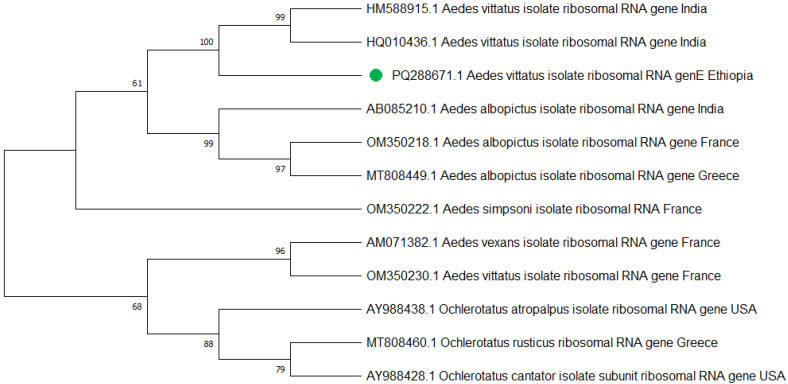
The phylogenetic tree representing the *Aedes vittatus* ITS1 gene of the Ethiopian sequence is indicated with a green color. The evolutionary history was inferred using the neighbor-joining method [40]. The bootstrap consensus tree inferred from 500 replicates [41] and the percentage of replicate trees in which the associated taxa clustered together in the bootstrap test (500 replicates) are shown next to the branches [41]. The evolutionary distances were computed using Tamura–Kumar method [42]. This analysis involved 13 nucleotide sequences. All positions containing gaps and missing data were eliminated. There was a total of 554 positions in the final dataset. Evolutionary analyses were conducted in MEGA11 [43].

Similarly, a total of 25 *Ae. vittatus*, which were identified according to their morphological features, were also confirmed to be *Ae. vittatus* using the PCR analysis targeting the internal transcribed spacer 1 (ITS1) gene (Figure 6). Three PCR products, one from each town, were sequenced, edited, and aligned using the BioEdit Sequence Alignment Editor and deposited in GenBank under the accession codes of *Aedes vittatus* isolate AEVIW2 (PQ288671.1).

## 4. Discussion

The genus *Culex* prevailed, followed by *Aedes*, *Anopheles*, and *Culiseta* in Genda-Wuha, Kokit, and Metema-Yohannes towns of the Metema district in northeastern Ethiopia during the period of June–September 2023. However, the genus *Culiseta* was not observed in Metema-Yohannes. Among the 16,324 mosquitoes caught by CDC-LT, 79.52% (12f492) were collected during the night hour and 23.41% (3822) during the daylight hour. All the *Anopheles*, *Culex*, and *Culiseta* were caught only during the night hours, whereas *Aedes* species were caught during both night and daylight hours. This entails that the Metema district was at a high risk of several types of mosquito-borne diseases, including arboviral diseases, filariasis and malaria, among others. *Anopheles* species were observed biting in the evening, although peak biting hours could vary with geographical setting and time, such as in the early morning [44,45], while *Culex* and *Culiseta* commonly bite overnight [46] and *Cx. quinquefasciatus* and *Cx. tritaeniorhynchus* exhibited unimodal nocturnal behavior in some instances [47].

*Aedes aegypti* exhibited higher density and higher biting rate outdoors than indoors during both the daylight hour and the night hour. In outdoors, *Ae. aegypti* exhibited two peak biting rates between 07:00 and 08:00 in the morning, followed by from 17:00 to 18:00 early night. The peak biting hours of *Ae. aegypti* matched with the active working hours of residents which could expose them to several viral disease infections. Similarly, its indoor peak biting rate in the morning could also expose households to infections as they carry out indoor activities. However, the indoor night biting rate of *Ae. Aegypti* can be prevented, provided that the households sleep under properly tucked mosquito nets. Similar studies reported two peak indoor biting activities; one in the morning and the other in the afternoon [29,48]. However, according to another research, *Ae. aegypti* exhibits a trimodal pattern of biting activity in the morning, midday, and afternoon [49]. Furthermore, the other authors propose that *Ae. aegypti* may have adapted to become diurnal by expanding its activity from nocturnal hours to both indoor and outdoor [50].

The morning and early night peak biting rate of *Ae. vittatus* also matched with the active working hours in the area. The peak outdoor biting hours of *Ae. vittatus* that aligned with the active working hours could expose the residents to viral disease infections. In a previous study, *Ae. vittatus* was reported to exhibit nocturnal biting rhythms in rural south India [51]. Speculated *Aedes* mosquitoes have an ecological adaptation that allows them to transition from nocturnal to diurnal biting behaviors, whose behavioral change mechanism is likely weak sunlight intensity after sunrise and before sunset [49,52]. Urban landscapes can influence biting times due to factors like artificial lighting and temperature variations. For instance, well-lit areas may extend mosquito activity into the night, altering traditional biting patterns.

*Aedes aegypti* and *Ae. vittatus* exhibited two peak biting times outdoors and indoors in common. This observation was similar to the earlier peak biting hours [49,53]. *Aedes aegypti* and *Ae. vittatus* are considered to be the major vectors of arboviruses such as dengue fever virus (DENV), yellow fever virus (YFV), chikungunya virus (CHIKV), and Zika virus (ZIKV), and hence they could be responsible vectors for chikungunya virus [31] and dengue virus [32] infections in the Metema district. The district is also a borderline with East Sudan, where there have been arboviral disease transmission and outbreaks of dengue fever [31,32,54]. Thus, the Metema district had a high risk of arbovirus transmission according to the Man–vector count, exceeding two female bites per hour was indicative of a significant risk of viral disease transmission [55,56].

Both *Ae. aegypti* and *Ae. vittatus* were observed most abundantly closer to humans, followed by cattle, sheep, goats, and donkeys. This indicates their preference to feed on humans and also their importance as disease vectors in the area. In a previous report, *Ae. aegypti* was also observed to feed on human blood most commonly [57]. The transmission potential of a vector-borne pathogen is influenced to a large extent by the human blood-feeding behavior of the vector [58,59].

The abdominal status of mosquitoes appears to be a critical aspect of field studies as it offers valuable data on feeding behavior, pathogen infection status, and ecological interactions. This information provides basic data to manage mosquito populations and the diseases they transmit. In the towns, the great majority of both *Ae. aegypti* and *Ae. vittatus* was observed to be unfed. These results are supported by a previous report in which case a high number of unfed *Ae. aegypti* were collected outdoors using sticky ovitraps [60]. The proportion of *Ae. aegypti* and *Ae. vittatus* with distended abdomens indicated their recent access to diverse blood meal sources, high reproductive potential, and also high-risk arboviral disease transmission in the towns.

Mitochondrial cytochrome oxidase subunit 1 (COI) and internal transcribed spacer 1 (ITS1) gene analysis provided a good molecular confirmation of *Ae. aegypti* and *Ae. vittatus* species identification, respectively. The subpopulation of *Ae. aegypti* and *Ae. vittatus* species’ nucleotide sequences at Metema-Yohannes, Kokit, and Genda-Wuha towns had no differentiated haplotype diversity in the study towns. However, more genomic data is needed for deeper phylogeographic analysis. *Aedes aegypti* haplotype sequences in the present study in northwest Ethiopia and in China and the UK had highly significant relationships in species as indicated by the GeneBank database. However, a high level of diversity variation is evidence of *Ae. aegypti* haplotypes in northwest Ethiopia compared to west Africa, south Africa, Saudi Arabia, and Brazil. Similarly, *Ae. vittatus* in northwest Ethiopia had nearly 100% per identity with India’s national nucleotide sequence of *Ae. vittatus* certified by the gene bank database. However, a phylogenetic tree revealed that there is a significant variation in haplotype diversity when compared to *Ae. vittatus* haplotypes from the United States, Greece, and France.

Overall, this phylogenetic tree indicates that the origin of the *Aedes* population in Ethiopia is more likely to be China and the UK for the *Ae. aegypti* species, while *Ae. vittatus* is highly related to India. The differentiation trends among Ethiopian samples from three towns suggest the absence of the subpopulations of *Ae. aegypti* and *Ae. vittatus* species among our collection in Ethiopia. However, we need to further explore the level of differentiation between subpopulations within Ethiopia and the differentiation between Ethiopia and neighboring countries.

### Limitations of the Study

The study on biting hours and host-seeking preferences of *Ae. aegypti and Ae. vittatus* was carried out in urban settings. Thus, further research should include rural areas in the district to come up with a better conclusion in relation to peak biting hours, blood meal source, species diversity, and arbovirus infection rates of *Ae. aegypti* and *Ae. vittatus*. In addition, the host-seeking behavior of *Ae. aegypti* and *Ae. vittatus* remains to be described using molecular tools. Molecular identification of subpopulation nucleotide (haplotype) diversity and subspecies of *Ae. aegypti* and *Ae. vittatus* was not performed due to financial constraints.

## 5. Conclusions

*Aedes aegypti* and *Ae. vittatus* exhibited peak biting activity during the early morning and late afternoon/early evening hours. The biting hours align with human activity patterns, increasing the likelihood of human-mosquito interactions and escalating the risk of disease transmission. The biting hours in an urban setting can be affected by entry number and late one-hour biting rate observed indoors rather than outdoors due to artificial lighting and temperature variations. For instance, well-lit areas may extend mosquito activity into the night, altering traditional biting patterns. It plays a role in the ecology of disease transmission, influencing host populations and dynamics within ecosystems. Mosquitoes adapt to human host-seeking patterns that indicate an evolutionary relationship; they may develop resistance to control measures, complicating management strategies. Future research should focus on rural and sylvatic arboviral vector distribution in large geographic areas and assessing the blood meal sources for a comprehensive assessment of arbovirus risk, and estimating the reservoir hosts of arbovirus should be required.

## Figures and Tables

**Table 1:** Mosquitoes collected during daylight hours and night hours in Metema District, Northwest Ethiopia from June to September 2023.

Study Town	Species	Day Light Time		Day Night Time		
		(6:00–18:00)		(18:00–6:00)		
		Indoor	Outdoor	Indoor	Outdoor	Total
Metema-Yohannes	*Aedes aegypti*	309	839	295	561	2004
	*Aedes vittatus*	147	380	133	184	844
	*Aedes albopictus*	17	19	0	12	48
	*Aedes communis*	0	0	0	0	0
	*Aedes opok*	13	9	0	6	28
	*Anopheles gambiae* s.l.	0	0	76	123	199
	*Anopheles stephensi*	0	0	37	65	102
	*Anopheles pretoriensis*	0	0	11	23	34
	*Anopheles christyi*	0	0	5	7	12
	*Anopheles cinereus*	0	0	3	6	9
	*Culiseta longiareolata*	0	0	0	0	0
	*Culex* spp.	0	0	1220	2415	3635
Total		486	1247	1780	3402	6915
Kokit	*Aedes aegypti*	163	494	107	396	1160
	*Aedes vittatus*	87	242	67	172	568
	*Aedes albopictus*	0	0	0	0	0
	*Aedes communis*	17	29	0	19	65
	*Aedes opok*	0	0	0	0	0
	*Anopheles gambiae s.l*.	0	0	42	107	149
	*Anopheles Stephensi*	0	0	15	26	41
	*Anopheles Pretoriensis*	0	0	3	7	10
	*Anopheles christyi*	0	0	0	5	5
	*Anopheles Cinereus*	0	0	0	3	3
	*Culiseta Longiareolata*	0	0	0	25	25
	*Culex* spp.	0	0	768	1152	1920
Total		267	765	1002	1912	3946
	*Aedes aegypti*	193	519	87	370	1169
	*Aedes vittatus*	87	256	28	112	483
	*Aedes albopictus*	0	0	0	0	0
	*Aedes communis*	0	12	13	17	42
Genda-Wuha	*Aedes opok*	0	0	0	0	0
	*Anopheles gambiae s.l*.	0	0	53	81	134
	*Anopheles Stephensi*	0	0	17	47	64
	*Anopheles Pretoriensis*	0	0	10	16	26
	*Anopheles*, *chrisityi*	0	0	0	7	7
	*Anopheles Cinereus*	0	0	0	5	5
	*Culiseta Longiareolata*	0	0	0	32	32
	*Culex* spp.	0	0	1341	2160	3501
	*Total*	280	787	1549	2847	5463

**Table 2:** Host-seeking behavior and physiological status of *Aedes aegypti* and *Aedes vittatus* in Metema district, Northwest Ethiopia, June–September 2023.

*Ae. aegypti*					*Ae. vittatus*			
	Unfed n (%)	Blood Fed n (%)	Gravid n (%)	Total N	Unfed n (%)	Blood Fed n (%)	Gravid n (%)	Total N
Human	3318 (81.8)	232 (86.2)	90 (80.4)	3640 (82.1)	1329 (74.5)	118 (79.2)	24 (55.8)	1471 (74.5)
Cattel	276 (6.9)	18 (6.7)	6 (5.3)	300 (6.7)	197 (11.6)	14 (9.4)	4 (9.3)	215 (10.9)
sheep	194 (4.9)	7 (2.6)	4 (3.6)	205 (4.6)	104 (6.1)	7 (4.7)	7 (16.3)	118 (6.0)
Goat	153 (3.9)	8 (2.9)	7 (6.2)	168 (3.8)	82 (4.8)	6 (4.0)	4 (9.3)	92 (4.7)
Donkey	114 (2.9)	4 (1.5)	5 (4.5)	123 (2.7)	71 (4.2)	4 (2.7)	4 (3.3)	79 (4.0)
Total	4055 (91.4)	269 (6.1)	112 (2.5)	4436	1783 (90.3)	149 (7.5)	43(2.2)	1975

## Data Availability

The data presented in this study are openly available in [Wondmeneh Jemberie Kassa] [10.3390/tropicalmed9030052] [https://orcid.org/0000-0002-7545-9890].

## Supplementary Materials

The following supporting information can be downloaded at: https://www.mdpi.com/article/10.3390/tropicalmed10020038/s1, Figure S1: Image of morphological characteristics: (**a**) *Ae. aegypti* species. (**b**) *Ae. vittatus* species.

## Abbreviations

YFV: Yellow fever virus; DENV: Dengue virus; CHIKV: Chikungunya virus; ZIKV: Zika virus; ANOVA: Analysis of variance; *Ae. aegypti*: *Aedes aegypti*; *Ae. vittatus*: *Aedes vittatus*; PCR: Polymerase chain reaction; COI: cytochrome oxidase subunit 1; ITS1: internal transcribed spacer 1.
