# Supplementary figures and images for: Biting Hour and Host Seeking Behavior of Aedes Species in Urban Settings, Metema District, Northwest Ethiopia
Source: Trop Med Infect Dis. 2025 Jan 28;10(2):38. doi: 10.3390/tropicalmed10020038 (PMC11860606; doi:10.3390/tropicalmed10020038)

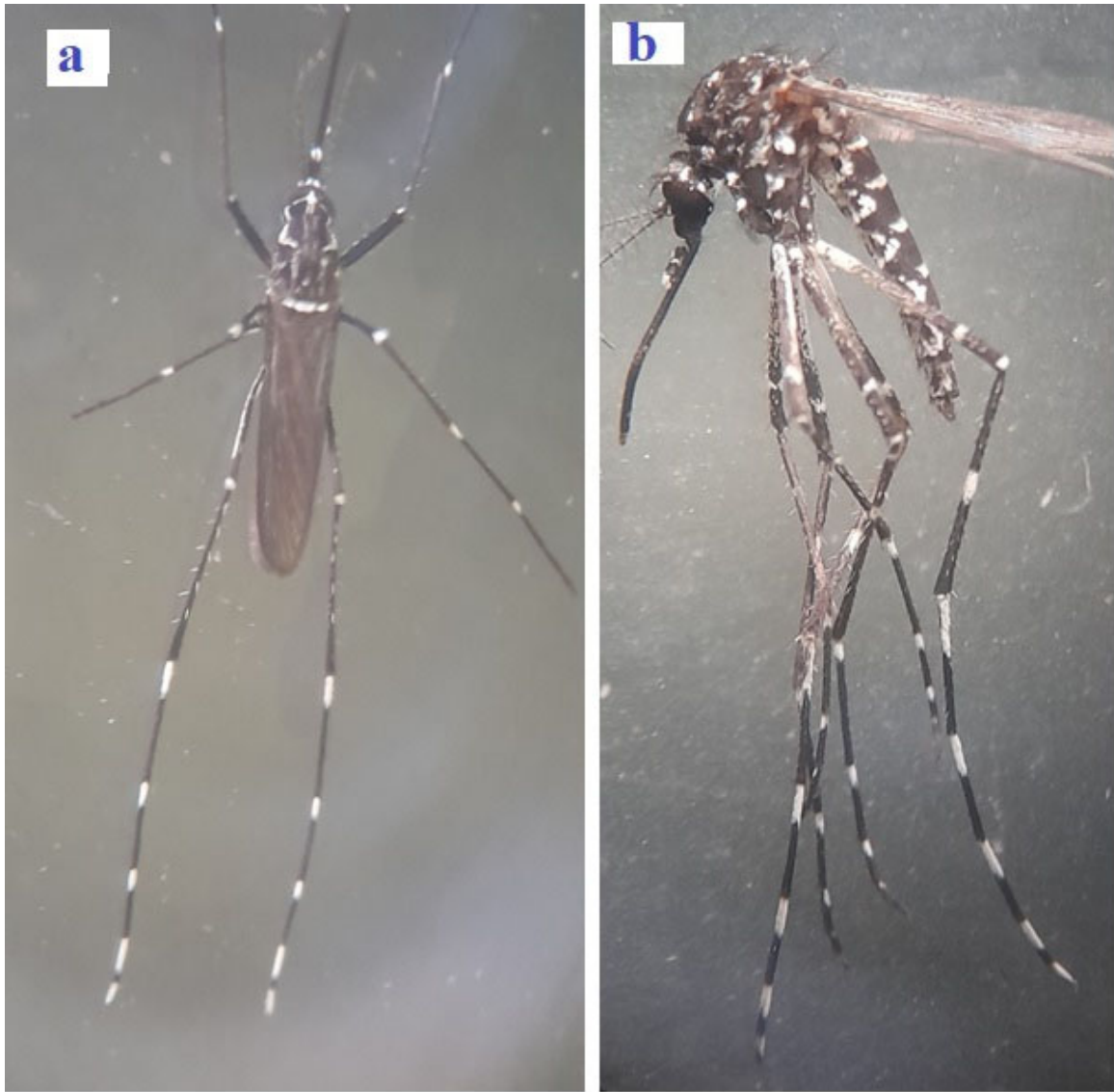

**Figure S1.** Image of morphological characteristics: (a) *Ae. aegypti* species. (b) *Ae. vittatus* species.

Supplement: Supplementary file 1 [file tropicalmed-10-00038-s001.zip › tropicalmed-3380988-supplementary.pdf]
